# Green algae and gelatine derived nitrogen rich carbon as an outstanding competitor to Pt loaded carbon catalysts

**DOI:** 10.1038/s41598-021-86507-5

**Published:** 2021-03-29

**Authors:** Anna Ilnicka, Malgorzata Skorupska, Magdalena Tyc, Kinga Kowalska, Piotr Kamedulski, Wojciech Zielinski, Jerzy P. Lukaszewicz

**Affiliations:** 1 Faculty of Chemistry, Nicolaus Copernicus University in Torun, Gagarina 7, 87-100 Torun, Poland; 2 Centre for Modern Interdisciplinary Technologies, Nicolaus Copernicus University in Torun, Wilenska 4, 87-100 Torun, Poland

**Keywords:** Chemistry, Materials science, Nanoscience and technology

## Abstract

The development of effective catalysts for the oxygen reduction reaction (ORR) is a significant challenge in energy conversion systems, e.g., Zn–air batteries. Herein, green-algae- and gelatine-derived porous, nitrogen-rich carbons were extensively investigated as electrode materials for electrochemical catalytic reactions. These carbon-based catalysts were designed and optimized to create a metal-free catalyst via templating, carbonization, and subsequent removal of the template. The additional incorporation of graphene improved electronic conductivity and enhanced the electrochemical catalytic reaction. Porous carbons with heteroatoms were used as effective platinum-free ORR electrocatalysts for energy conversion; the presence of nitrogen in the carbon provided more active sites for ORR. Our catalyst also displayed notable durability in a rechargeable Zn–air battery energy system. More importantly, the nitrogen-containing porous carbons were found to have comparable ORR performance in alkaline media to commercially available electrocatalysts. The manuscript demonstrates that nitrogen atom insertion is an appropriate approach when aiming to eliminate noble metals from the synthesis route. N-doped carbons are competitive materials compared to reference platinum-based catalysts.

## Introduction

Metal–air batteries, mainly Li–, Zn–, Al–, and Mg–air batteries, possess theoretical and practical energy densities^1–3^ several times higher than those of the very popular Li-ion batteries. Because they possess very high durability and fewer operational safety^4^ issues, Zn–air batteries have attracted more attention regarding their use in a wide spectrum of energy conversion systems than, e.g., Li–air batteries have. The operational environment for Zn–air batteries is a neutral electrolyte or aqueous alkaline, increasing their environmental safety^1,5,6^. Platinum-based electrocatalysts are widely used due to their relatively low overvoltage and high current density^5,7,8^; however, they have their disadvantages, such as no resistance to intermediates, slow kinetics, and instability with prolonged use^9^. Moreover, platinum-loaded electrode materials for anode design do not meet the cost and environmental requirements of the technologies of tomorrow. For example, platinum requires recycling due to its cost. An alternative would be the use of active carbon nanofibers^10^ and carbon nanofibers doped with Co, Fe, and N^11–13^. Other carbon-based nanomaterials, such as nanotubes and graphene, are also under intensive investigation as potential replacements for platinum which would reduce costs and increase stability^14,15^. Shui et al. show that metal-free, nitrogen-doped carbon nanotubes and their graphene composites exhibit significantly better long-term stability in ORR^16^. Wei et al. also used nitrogen-doped nanotubes and doped graphene which showed a transferred electron number equal 3.6–4.0^17^. However, these carbons were obtained by the chemical vapor deposition (CVD) method, which is relatively complex due to using an environmentally unfriendly Ni catalyst surface and gas mixtures of NH_3_, H_2_, Ar, and CH_4_. The papers cited as well as many others prove that nitrogen-doped activated carbons obtained from synthetic and natural carbon precursors can also be an alternative to expensive Pt catalysts. Lately, more and more attention is being paid to natural precursors for N-rich activated carbons. An example of this research trend is the use of fish bones to obtain micro- and meso-porous carbon materials active in ORR^18^. In another case, the catalyst for ORR was a multiphase nanomaterial obtained from gelatine that had been treated with iron and magnesium nitrate or Ketjenblack^19,20^. However, it is still a real challenge to obtain porous, heteroatom-doped carbon catalysts with a high specific surface area that would better facilitate electrolyte transport. So far, templates like the NaCl sacrificial template^21^, CaCl_2_^22^, and SiC^23^ have been used to produce catalysts from gelatine. Authors assume that algae can also be a very promising carbon precursor due to their common accessibility and high nitrogen content. Some studies seem to confirm this assumption, for example, the case in which this natural precursor was activated using KOH, though the resulting specific surface area was small. In another example, algae were treated by a hydrothermal method to obtain N-doped carbon (but nonporous)^24^.

Despite reports already published, a facile synthesis of novel N-doped catalysts with good conductivity, and especially with a unique porous architecture, is still a challenge. Consequently, it is essential to explore an approach to this sort of facile method to produce novel, N-rich, hierarchical porous carbons from algae and gelatine, with a unique three-dimensional (3D) structure, high specific surface area, and excellent catalytic activity for ORR.

The current work addresses the research challenges mentioned above, i.e., a metal-free catalyst for oxygen reduction reactions in an alkaline medium was synthesized through the carbonization of green algae and gelatine. These natural precursors were selected due to the content of nitrogen-rich peptides, which were the assumed source of nitrogen atoms embedded in the carbon matrix after thermal degradation. In the case of *Chlorella vulgaris,* some phospholipids are expected to be present as well. However, the lipids do not contain considerable amounts of nitrogen which could be transferred to the carbon matrix. The authors investigate catalytic performance by using widely approved cyclic voltammetry (CV) and rotating disc electrode (RDE) techniques. The most important motivation is to discover, investigate, and present attractive properties of the N-rich porous carbons being produced, as exhibited in comparison to a commercial Pt/C electrocatalyst, commonly regarded as an excellent cathode catalyst for ORR. The developed method can provide simple but efficient, versatile approaches to low-cost production of N-rich porous carbons to be used as efficient, metal-free electrocatalysts for metal–air batteries.

## Results and discussion

### Material characterisation

**Figure 1:**
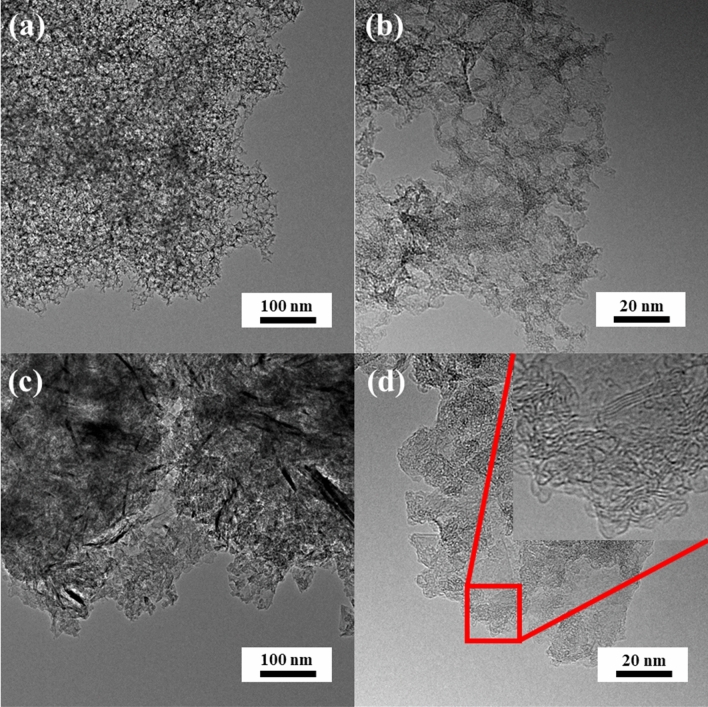
HRTEM images of (**a**,**b**) N-GPC-800 and (**c**,**d**) N-GPC-GR-800.

**Figure 2:**
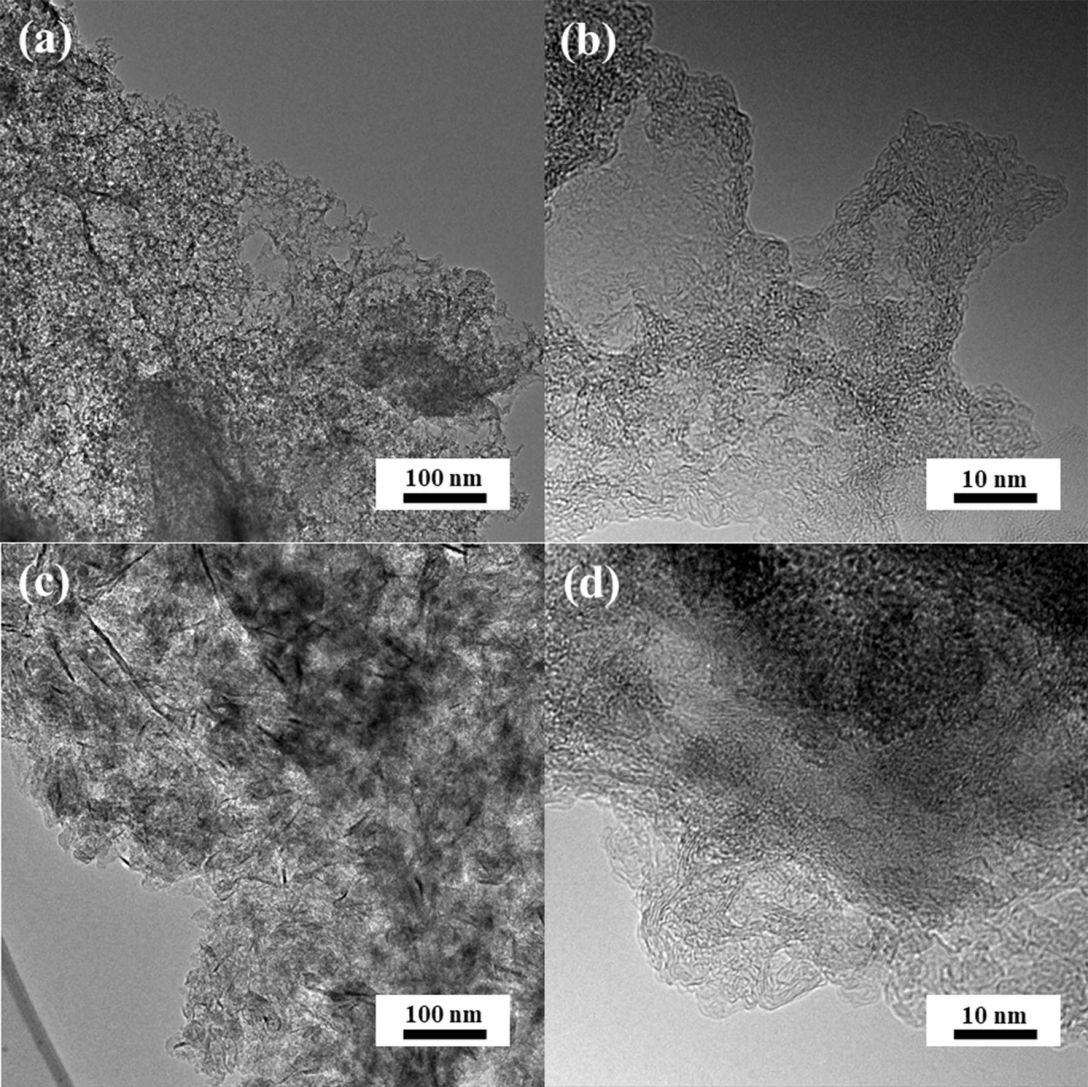
HRTEM images of (**a**,**b**) N-APC-800, (**c**,**d**) N-APC-GR-800.

It was decided that algae-derived activated carbons would be investigated in detail, since there is a lack of complex information on their properties. The samples’ morphology was characterized first. High-resolution transmission electron microscopy (HRTEM) images of gelatine- and green-algae-derived carbon samples reveal a three-dimensional “spongy” structure. All samples in series N-GPC-T, N-GPC-GR-T, N-APC-T, and N-APC-GR-T show a hierarchical structure containing mesopores. HRTEM images reveal a majority of the pores that is spherical with a diameter of approximately 20 nm; these observations show geometry and size representative of the silica particles used as a template during synthesis (Fig. 1a,b). The width of graphene nanoribbons (Fig. 1c,d) was less than 20 nm (typically 10 nm), whereas the thickness was less than 2.5 nm, corresponding to about eight layers of graphene sheets or fewer. The N-APC-800 and N-APC-GR-800 carbons (Fig. 2) possessed little long-range order and no discernible crystallite structure. Moreover, the transmission microscopy images attest to a uniform 3D structure of interconnected network nanoarchitecture (Fig. 2a,b). Microscopic observations do not determine all aspects of the obtained materials’ pore structure. The presence and considerable share of mesopores was assumed to be a key feature expected from the proposed synthesis manner.

**Figure 3:**
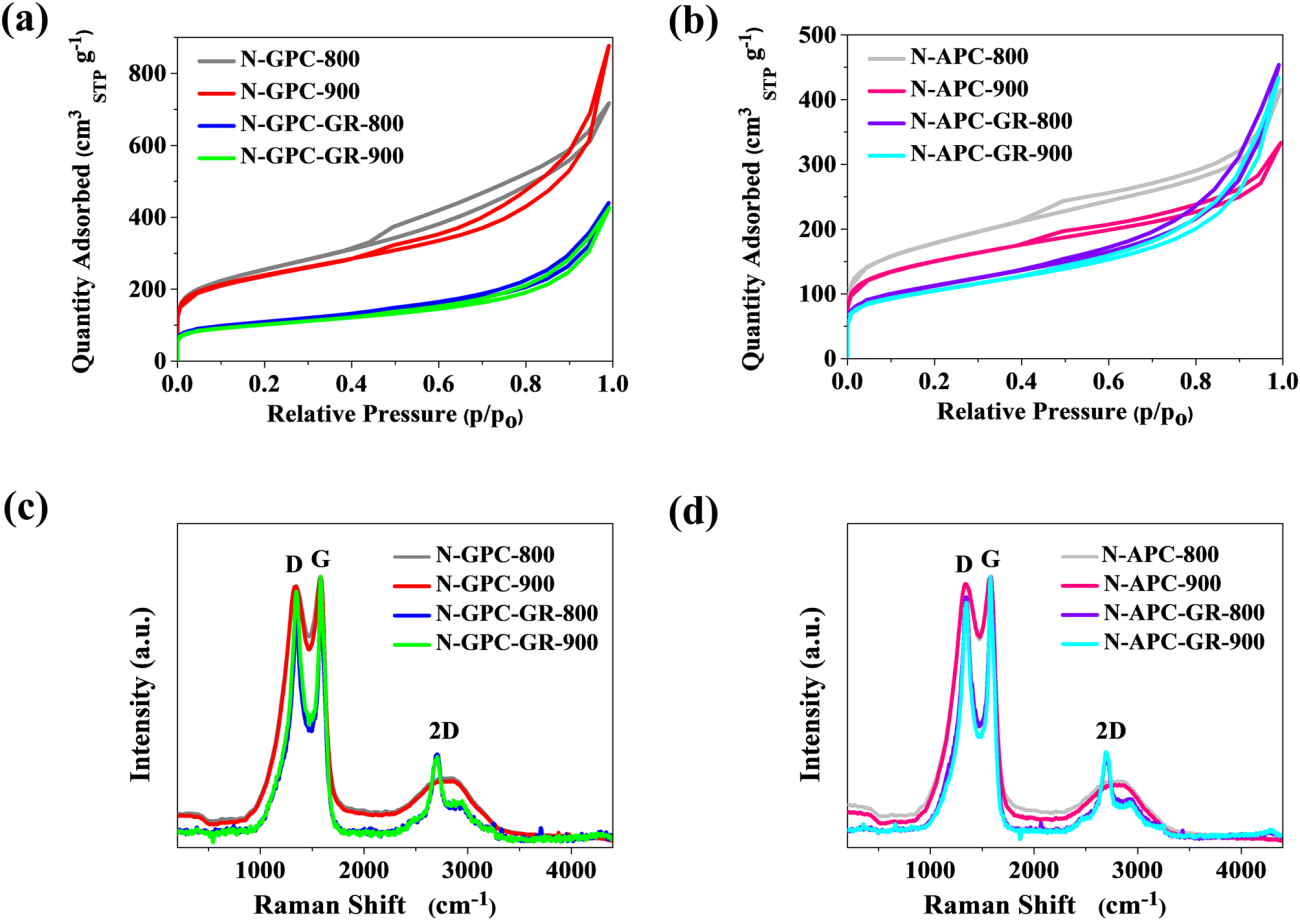
(**a**,**b**) Nitrogen adsorption–desorption isotherms, (**c**,**d**) Raman spectra for gelatine- and green-algae-derived carbons.

Figure 3a,b show the N_2_ nitrogen adsorption–desorption isotherms of the nitrogen-rich carbon materials obtained from, respectively, gelatine or green-algae. The mesoporous nature of the material is evident from the type IV isotherm (using the IUPAC classification), i.e., the visible hysteresis loop in the P/P_0_ = 0.4 to 1.0 range, indicating the existence of abundant mesopores in the N-GPC-T series. According to pore size distribution analysis, the samples’ pore size distribution was in the microporous (ca. 0.52, 1.44, and 1.76 nm) and mesoporous regions (2.75, 3.16, 3.76, and 5.88 nm). Specific surface area decreased with increasing carbonization temperature, which may be linked to the carbon framework collapsing, leading to the vanishing of open pores. These results indicate that the N-GPC-T series exhibited a higher specific surface area and total pore volume compared to carbons in the N-GPC-GR-T series, obtained with graphene. The pore sizes in all green-algae-derived porous carbons of all series were in the same ranges, predominantly in two regions, similar to samples obtained using gelatine: micropores centred in the 0.52–1.76 nm region and mesopores centred in the 2.75–5.88 nm region. A decrease of specific surface area could be observed again as the pyrolysis temperature reached 900 °C. The 3D porous N-rich carbon possessed abundant edges, thin walls, and conductive networks which could facilitate fast transportation of mass and charge, consequently facilitating electrochemical reactions.

In general, carbon electrode materials are expected to show a large surface area, since the surface layer is where electrochemical phenomena occur. A Brunauer–Emmett–Teller (BET) analysis was used to examine the difference of surface area between gelatine- and green-algae-derived porous carbons. Pore parameters were extracted from the isotherms and are listed in Table 1. The gelatine-derived carbon material N-GPC-800, carbonized in a nitrogen atmosphere at 800 °C, exhibited the highest specific surface area (S_BET_) of 880 m^2^ g^−1^, as well as a total pore volume (V_t_) equal 1.12 cm^3^ g^−1^, higher than that of samples obtained at 900 °C. A similar tendency towards the decrease of S_BET_ was observed for samples N-APC-800 and N-APC-900, their S_BET_ equal to 623 and 532 m^2^ g^−1^, respectively. No significant changes in the surface area were evident for samples obtained with graphene, whereas for N-GPC-GR-800 and N-GPC-GR-900 the S_BET_ came to 384 and 360 m^2^ g^−1^, respectively. In summary, appropriate surface area values are achievable by means of the proposed synthesis pathways, though not all manufacturing scenarios are equivalent regarding the ease of improving those values. N-GPC and N-APC protocols with no graphene addition attained a better developed surface area.

**Table 1:** Physicochemical properties and elemental composition via bulk combustion of the gelatine and green algae-derived carbons.

Carbon sample	Elemental content (wt.%)			S_BET_ (m^2^ g^−1^)	V_t_ (cm^3^ g^−1^)	V_mi_ (cm^3^ g^−1^)	V_me_ (cm^3^ g^−1^)
	C	H	N				
N-GPC-800	68.28	2.19	10.08	880	1.12	0.27	0.85
N-GPC-900	73.89	1.09	7.41	842	1.39	0.25	1.14
N-GPC-GR-800	91.94	1.05	3.22	384	0.69	0.10	0.59
N-GPC-GR-900	90.79	1.02	3.24	360	0.67	0.10	0.57
N-APC-800	65.60	2.01	7.09	623	0.64	0.23	0.42
N-APC-900	64.92	1.88	5.74	532	0.51	0.19	0.32
N-APC-GR-800	88.81	1.38	2.26	391	0.72	0.10	0.61
N-APC-GR-900	90.79	0.90	2.16	366	0.68	0.10	0.58

Aside from the improvement of surface area and tailoring the pore structure towards a high mesopore content, the additional presence of nitrogen atoms was very much welcome, since N-containing surface functional groups are the potential catalytic centres responsible for oxygen reduction. Combustion elemental analysis was employed to investigate the elemental compositions of the gelatine- and green-algae-derived carbons. Nitrogen doping was observed and seen to be strongly influenced by three factors: type of precursor used, carbonization temperature, and presence or absence of graphene during synthesis. According to the elemental analysis results shown in Table 1, the samples synthesized without graphene (GF) contained ca. 64–73 wt.% of carbon, while those with graphene contained 88–91 wt.%. The highest nitrogen content for gelatine- and green algae-derived carbon samples obtained without graphene was 10.08 wt.% for N-GPC-800 and 7.09 wt.% for N-APC-800. For samples obtained with graphene, the bulk nitrogen content was still appreciable; at 800 °C the content of nitrogen was 3.22 wt.% for N-GPC-GR-800 and 2.26 wt.% for N-APC-GR-800.

To further compare the influence of the precursor and carbonization process on the structure of N-rich porous carbons and graphene, a Raman spectroscopic analysis at up to 4500 cm^−1^ was applied. Carbon microstructure provides insight into potential electrochemical catalyst application. The Raman shifts of the D-band and G-band for N-rich PC (Fig. 3c,d) were at approximately 1344 cm^−1^ and 1580 cm^−1^, respectively. The G band for N-rich PC with graphene shifted to 1346 cm^−1^. The degree of graphitization was appraised using Raman spectra to calculate the I_D_/I_G_ ratio; results indicate that the low degree of graphitization in the N-GPC-T and N-APC-T series could be attributed to defects caused by the presence of N-species. The Raman spectra also display small increases in the intensity ratio of the D and G bands (I_D_/I_G_) where carbonization temperature was increased. The observed trends indicate carbon defects caused by the integration of heteroatom species into the carbon framework. The continuing slight increase in the I_D_/I_G_ ratio until a carbonization temperature of 900 °C was reached may indicate the existence of more in-plane heteroatoms (graphitic-like); indeed, this is confirmed to be the case by the X-ray photoelectron spectroscopy (XPS) results presented in Table 2. The I_D_/I_G_ values (0.96 and 0.97) prove that the N-GPC-T series possessed defects on the carbon structure which are usually associated with a more disordered carbon structure. A slightly smaller value of I_D_/I_G_ in the N-GPC-GR-T series can be attributed to the presence of graphene sheets in the carbon structure. Raman results show that pristine, N-rich carbons tended to decrease the G band, implying that at the edge plane the degree of disorder with decreasing edge planes was discontinued. Furthermore, an additional sharp band ca. 2709 cm^−1^ (2D-band) was observed on the Raman spectrum, which is characteristic of a graphene structure. The presence of a broad and weak 2D band is a representative feature of graphene sheets distributed randomly rather than in distinct stacks.

**Table 2:** Surface composition of the gelatine- and green-algae-derived carbons derived via XPS.

Carbon sample	C (at.%)	O (at.%)	N (at.%)	Nitrogen forms (%)	
				N-5	N-Q
N-GPC-800	86.6	5.9	7.7	3.1	4.6
N-GPC-900	85.3	8.5	4.7	1.7	3
N-GPC-GR-800	94.2	2.4	3.5	1.6	1.9
N-GPC-GR-900	94.5	2.5	3.1	1.4	1.7
N-APC-800	86.3	6.9	6.2	2.5	3.7
N-APC-900	85.6	7.8	4.8	1.9	2.9
N-APC-GR-800	94.9	3.5	1.7	0.6	1.1
N-APC-GR-900	95.3	2.8	1.6	0.7	0.9

**Figure 4:**
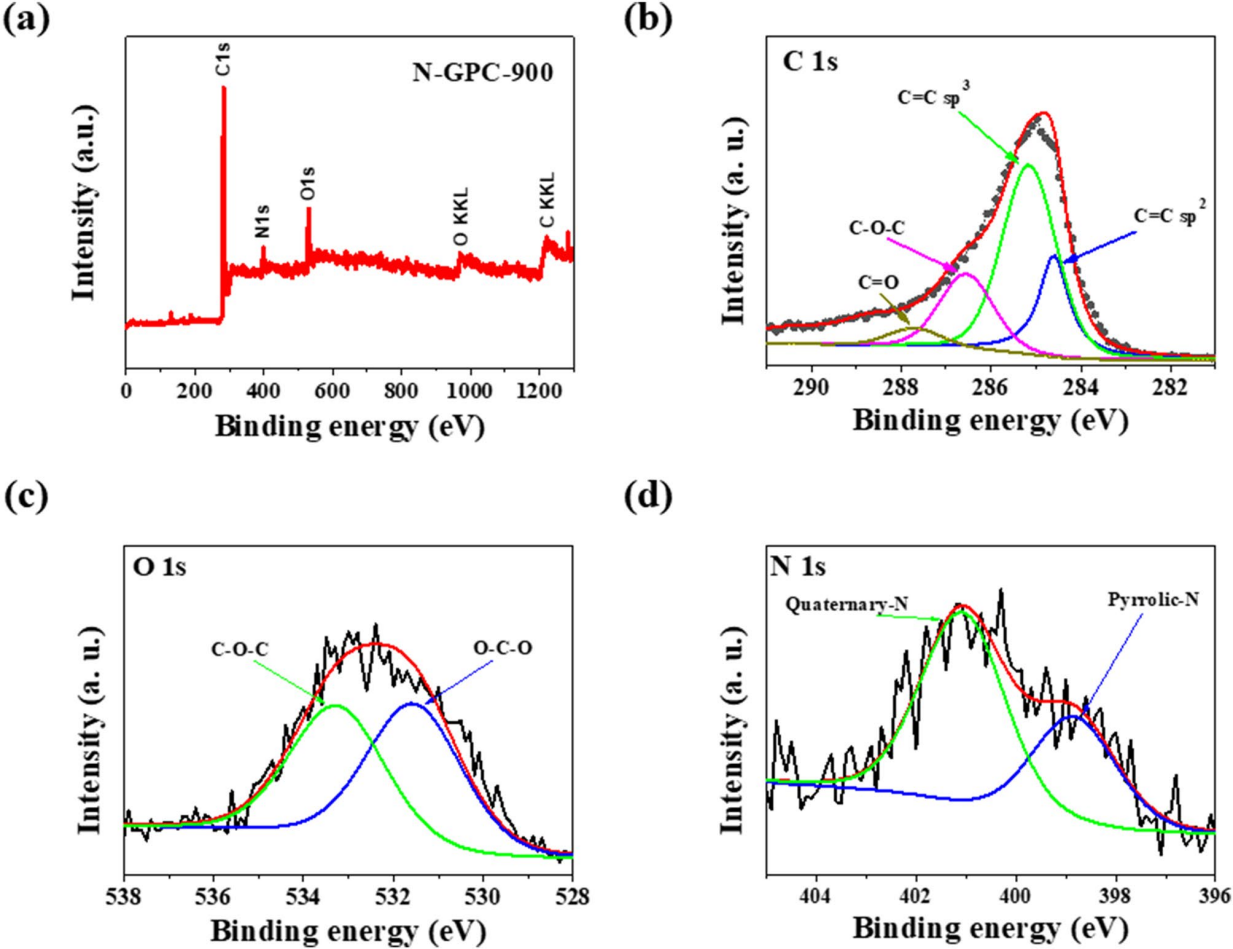
(**a**) XPS survey spectrum, high-resolution XPS of (**b**) C1s, (**c**) O1s, and (**d**) N1s spectra for N-GPC-900.

Combustion elemental analysis can be used to determine the elemental composition of the surface layer, as it provides information on the bulk content of some elements. Therefore, XPS measurements were performed to quantify the elemental composition and study the binding state of surface groups on N-rich porous carbons. As shown in Fig. 4a, the survey spectrum contains three main peaks, which correspond to carbon, nitrogen, and oxygen. The main elemental contents are qualified and listed in Table 2. A common practice is the deconvolution of XPS spectra to find particular surface species in the samples under investigation. Processing an example’s C1s spectrum (Fig. 4b) makes it possible to determine four peaks ascribable to the signals of graphitic sp^2^ bonded carbon (284.6 eV), sp^3^ bonded carbon (285.0 eV), C–O and C–N functionalities (286.4 eV), carbonyl groups (287.7 eV), and carboxyl groups (288.6 eV). The presence of a graphitic C=C bond beside C–C and C–O bonds justifies assumptions of the obtained N-doped porous carbons’ advanced electric conductivity. The existence of N–C bonds in C=N form proves that N was doped successfully into the porous carbon matrix. The content of C–C=C bonding systems permanently increased with the carbonization temperature growing from 800 to 900 °C. This result is consistent with the Raman analysis, indicating a positive influence of rising carbonization temperature on the degree of graphitization. The high resolution C1s spectrum in Fig. 4b shows predominant development of graphitic C=C bonds, along with other minor C–C and C–O bonds, implying good conductivity of synthesized N-GPC-900. Further, the two peaks at 531.6 and 533.2 eV in the O1s peak (Fig. 4c) are associated with single and double bonds between oxygen and carbon. The XPS N1s spectrum (Fig. 4d) was deconvoluted to pyrrolic nitrogen (N-5) and quaternary (graphitic) nitrogen (N–Q); these two peaks were centred at 399.1 eV and 400.8 eV, connected with high ORR activity^25–27^. Quaternary nitrogen groups dominated the concentration of nitrogen species in all N-rich porous carbons, as shown in Table 2. It was found that both the pyrolysis temperature and the addition of graphene had a significant effect on nitrogen content. The concentration of surface nitrogen was lower in XPS measurements than in elemental analysis, as surface nitrogen is easier to remove during thermal treatment. A high carbonization temperature range, i.e., 800–900 °C, was recognized to be indispensable for ensuring high electric conductivity, which is essential in electrode manufacturing. There was a negative effect in the form of surface species decomposition, which included nitrogen functional groups. The nitrogen elemental content decreased monotonically from 7.7 to 4.7 wt.% when carbonization temperature rose from 800 to 900 °C. We assume this was the effect of a more intensive decay of nitrogen-containing functional groups at higher temperatures. An in-depth analysis of C1s, N1s, and O1s reveals that surface species in the final samples differ significantly and the proportions in heteroatom bonding depend on the type of carbon precursor and carbonization temperature. Increased carbonization temperatures caused the nitrogen and oxygen content to reduce. This effect leads to the expectation of thermal instability in nitrogen dopants in the porous carbon structure. However, sample N-GPC-900 should still be a good candidate for a metal-free electrocatalyst in ORR due to having a high surface area, developed porosity, and high graphitization degree.

### Electrochemical performance

**Figure 5:**
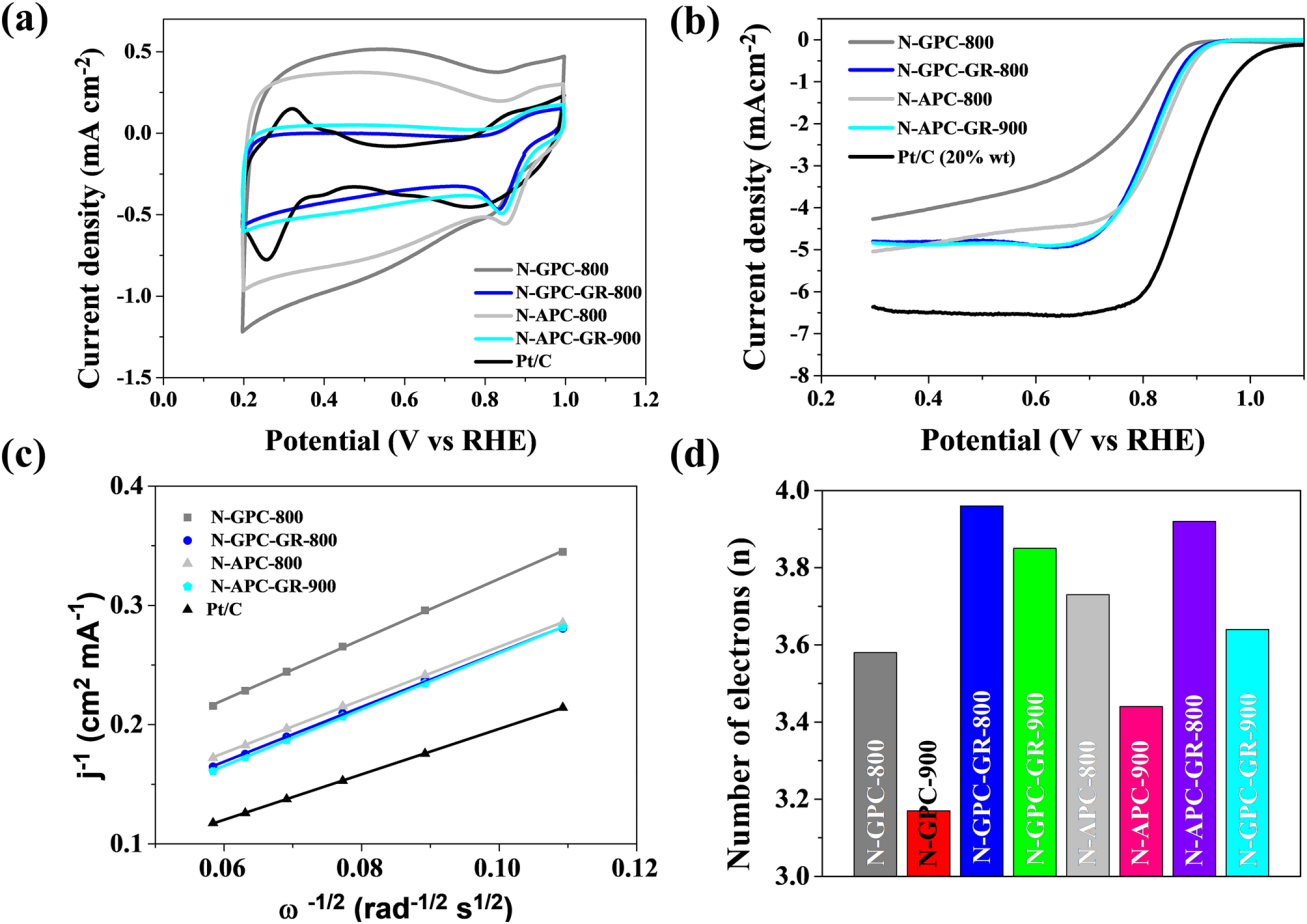
Results of catalyst activity in the oxygen reduction reaction for N-rich gelatine-derived carbons and the Pt/C catalyst. (**a**) CV curves of electrocatalysts in an O_2_-saturated 0.1 M KOH solution for samples N-GPC-800, N-GPC-900; (**b**) LSV of various electrocatalysts on RDE measured at a scanning speed of 5 mV s^−1^ with a rotation speed of 1600 rpm in an O_2_-saturated 0.1 M KOH solution; (**c**) onset potential calculated on the basis of LSV curves; (**d**) Koutecky–Levich plot at 0.5 V.

The synthesis of N-rich activated carbon was performed with the aim of its application as an anodic material in ORR within certain electrochemical devices, like air-zinc batteries. The aptitude of investigated materials towards effective ORR was proven through electrochemical studies. We employed CV and linear sweep voltammogram (LSV) methodologies. The number of electrons active in the reduction of an oxygen molecule should approach 4, just as in the Pt-loaded paternal electrode material provided by Sigma Aldrich. Voltammograms measured in a 0.1 M KOH O_2_-saturated electrolyte as catalyst exhibit a well-defined cathodic peak (Fig. 5a), also observed for N-rich porous carbons and the commercial Pt/C catalyst (Fig. 5a). The cathode peak is visible at 0.81 V vs RHE for samples N-GPC-800 and N-GPC-900. The LSV curves (Fig. 5b) for the produced catalysts show a similar current density to the commercial Pt/C catalyst, and for samples N-GPC-800 and N-GPC-900 are equal 3.67 mA cm^−2^ and 4.56 mA cm^−2^, respectively. The N-GPC-900 sample exhibited a current density most similar to the reference Pt/C catalyst, measured at 6.34 mA cm^−2^. This was due to a high carbonization temperature, which influenced the formation of appropriate nitrogen functional groups and increased the conductivity of the material. It must be stressed that the cathodic performance of Pt/C catalysts was met by N-modified carbons in which no Pt was present. To further investigate the ORR activity parameters, linear sweep voltammetry measurements were performed on RDE, at rotations between 800 and 2800 rpm, in an O_2_-saturated 0.1 M KOH. The ORR onset potentials of each sample were acquired from a RDE linear sweep at 1600 rpm (Fig. 5b). The values of the onset potential (Fig. 5c) for the N-GPC-800 and N-GPC-900 catalysts were similar and equal 0.88 V and 0.86 V, respectively. The Koutecky–Levich plots (Fig. 5d) for each catalyst were obtained from LSV at various rotational speeds; all lines showed good linearity. The electron transfer number, calculated from the slope of Koutecky–Levich plots for an electrode potential at 0.5 V, was in the range of 3.17 to 3.96 (Table 3). The highest n values were noted for N-GPC-GR-900 and N-GPC-GR-800, equal 3.85 and 3.96, respectively. These numbers confirm that catalytic activity was remarkably close or equal to the 4-electron ORR pathway of the commercial Pt/C catalyst. These results indicate a synergetic effect between a high specific surface area, nitrogen groups (possibly located at the carbon matrix edges), and a curved structure of N-rich porous carbons. In this research, carbons synthesized at 900 °C exhibited better catalytic properties than those synthesized at 800 °C. The highly porous structure of the N-rich materials allowed the alkaline electrolyte free access into its gaps, making the metal-free material an excellent catalyst for use in metal–air batteries or fuel cells. The findings above indicate that the chemical configuration of nitrogen may play a key role in determining the mechanism, though it’s nitrogen-active carbon atoms that are the actual active sites.

**Table 3:** ORR performance parameters of green-algae- and gelatine-derived porous, nitrogen-rich carbons and commercial Pt/C catalyst tested in alkaline media. E_p_—the peak potential, E_onset_—the onset potential, E_1/2_—the half wave potential, n—the number of transferred electrons.

Catalysts	E_p_ (V vs RHE)	E _onset_ (V vs RHE)	E_1/2_ (V vs RHE)	Diffusion-limiting current density (mA cm^-2^)	n (0.5 V)
N-GPC-800	0.81	0.88	0.78	3.67	3.58
N-GPC-900	0.81	0.86	0.76	4.56	3.17
N-GPC-GR-800	0.83	0.89	0.81	4.82	3.96
N-GPC-GR-900	0.83	0.90	0.82	4.58	3.85
N-APC-800	0.87	0.88	0.84	4.42	3.73
N-APC-900	0.82	0.89	0.80	3.92	3.44
N-APC-GR-800	0.82	0.88	0.80	4.24	3.69
N-APC-GR-900	0.84	0.90	0.82	4.89	3.84

**Figure 6:**
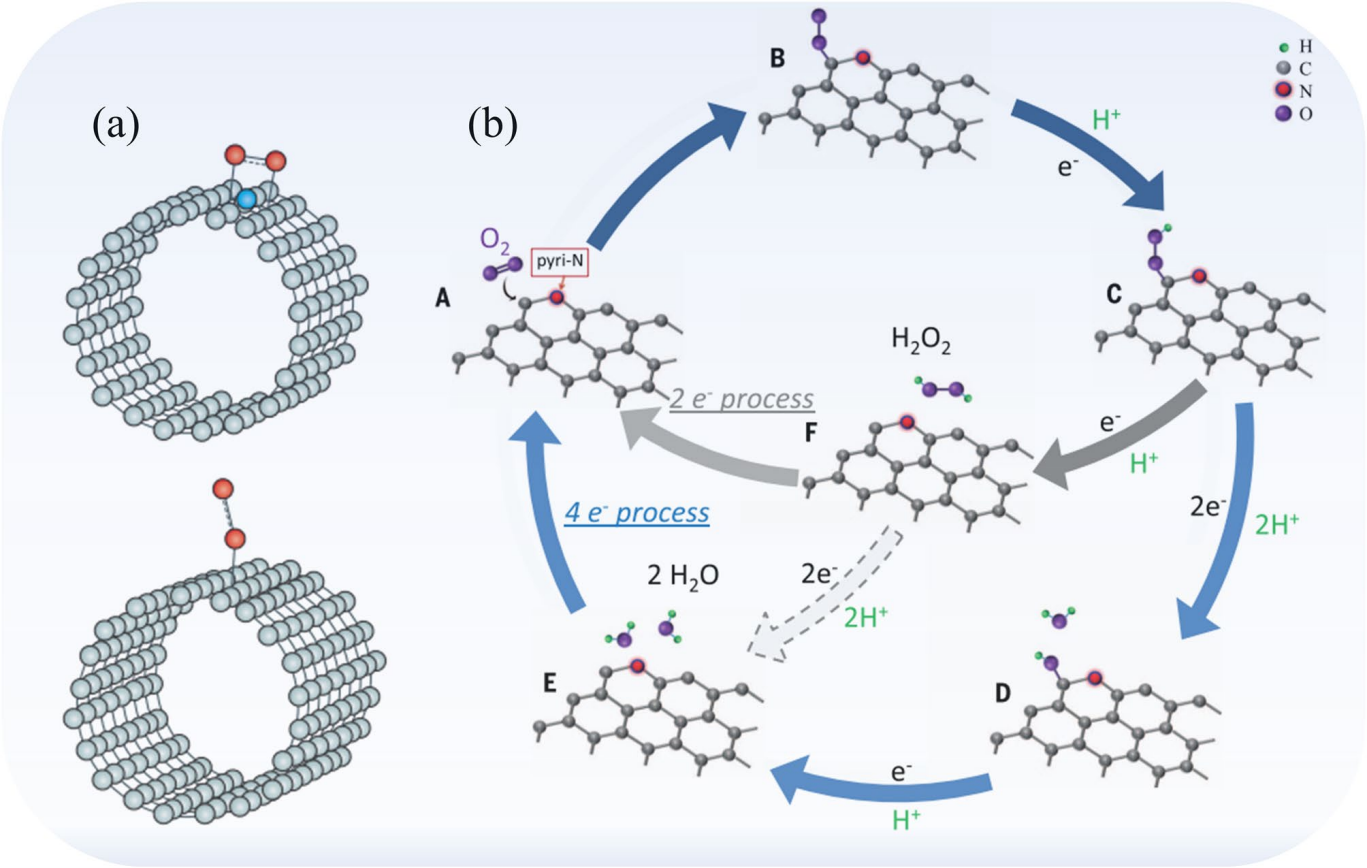
Two possible mechanisms of O_2_ adsorption on a N-doped CNT (**a**) after Liu et al.^29^. Two alternative pathways for ORR N-doped carbon graphitized materials (**b**) after Guo et al.^30^ (reproduced with permission from^28^). Copyright 2019, Nature.

As mentioned earlier, the search for effective non-metal catalysts for ORR is a mainstream pursuit in the field of electrochemical energy devices. The most common and effective, but traditional, ORR catalysts are noble metal based. In general, as demonstrated in this study by the comparative Pt/C catalyst, these electrode materials are capable of the ideal, 4-electron reduction of an O_2_ molecule. This material, as well as others based on metal, are out of the scope of interest for the current study, hence only N-doped carbon materials are discussed further. The mechanisms of ORR as it proceeds in N-doped graphitized carbons have been examined intensively. One of the latest studies was published by Ma et al.^28^ and Fig. 6 presents the two ORR mechanism options described therein.

Wei et al. reviewed numerous N-rich graphitized carbon materials working as ORR catalysts^17^. The comparative data from this research were assembled in Table 4 and from there it can be concluded that there is no direct correlation between nitrogen content and the transferred electron number. Additionally, there is no correlation between manufacturing protocol and ORR activity, for which the transferred electron number is a kind of primary indicator. Manufacturing protocols involve such different approaches as the pyrolysis of graphene oxide and polydopamine, CVD growth of graphene, and post-doping with a solid N precursor of graphitic C_3_N_4_. Regardless, the n values above 3.5 for N-rich graphitized carbons are regarded as outstanding. In the case of the current study, transferred electron number values for 6 of 8 investigated samples are situated above the limit of 3.5. The transferred electron number value for one of the samples is practically equal to the highest theoretical value that is 4. This underscores the importance of the current study, the achieved n values and the ease of manufacturing. The proposed synthesis method is competitive to others presented in the literature, while often also being simpler.

**Table 4:** Comparative data on the electron transfer number in ORR for diversified N-rich graphitized carbons.

N-content (at.%)	Electron transfer number (n)	References
25	3.2–3.7	^31^
10	3.4–3.6	^32^
1.6	2.1–3.9	^33^
2.0–2.7	3.5–4.0	^34^
26.1	3.2–4.0	^35^
2.4	3.8–3.9	^36^
2.78–3.79	3.89	^37^
0.52	3.2–3.9	^38^
12.5	3.69	^14^

### Rechargeable Zn–air battery tests

**Figure 7:**
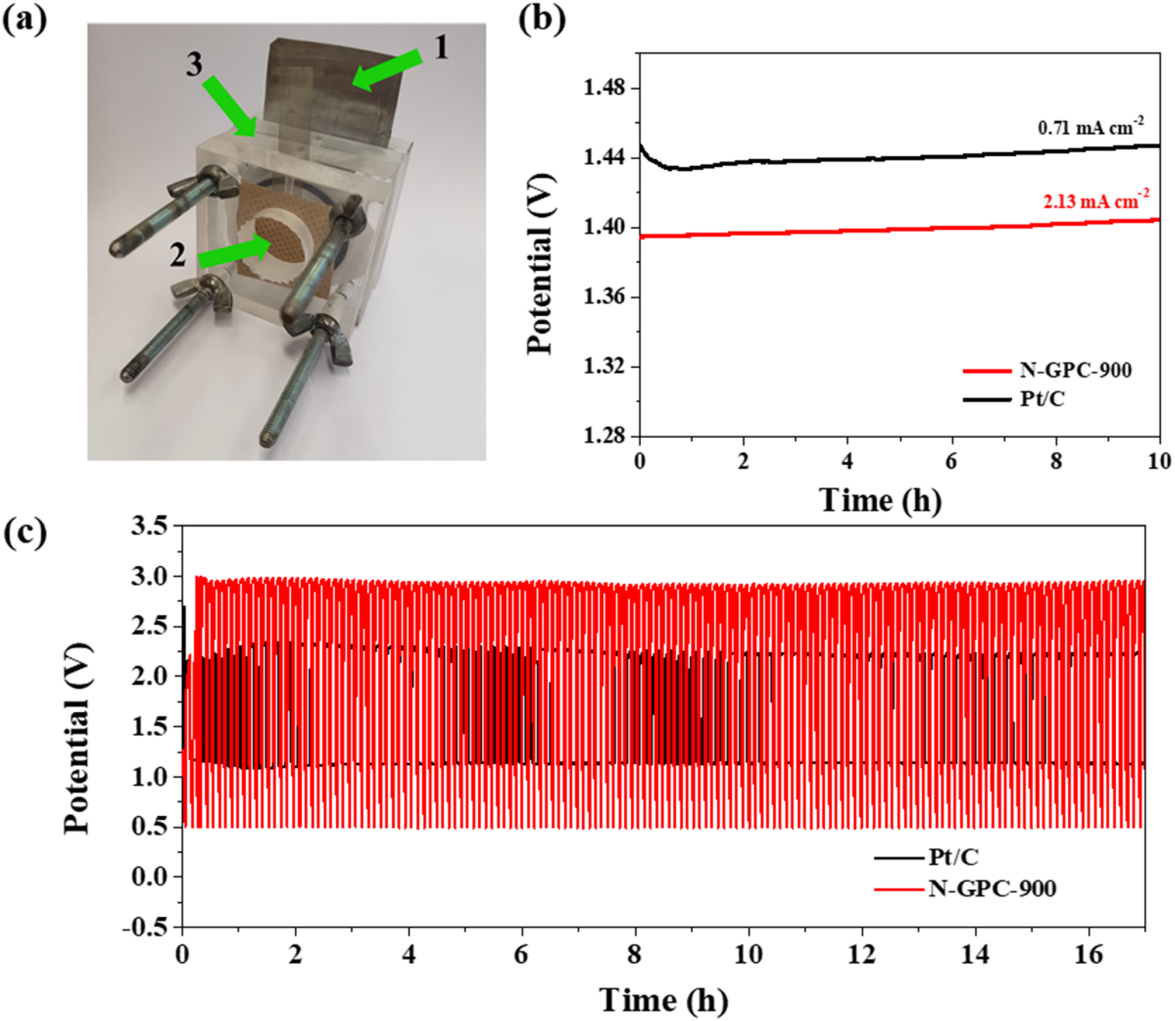
(**a**) Photograph of the rechargeable Zn–air battery where: (1) Zn-plate, (2) carbon paper with catalyst and separator, (3) place for electrolyte; (**b**) Galvanostatic discharge curves for N-GPC-900 and Pt/C catalyst at 0.74 mA cm^−2^ and 2.13 mA cm^−2^, respectively; (**c**) Galvanostatic discharge–charge cycling curves of Zn–air batteries with N-GPC-900 and Pt/C as catalyst.

To evaluate the practical application of the N-rich porous carbons, a home-built Zn–air battery was assembled using an N-rich catalyst as the air cathode and a zinc plate as the anode (Fig. 7a). The open-circuit potential (OCP) of the Zn–air battery with either N-GPC-900 or Pt/C catalysts as air-cathodes was determined to be 1.40 V and 1.45 V, respectively (Fig. 7b). Figure 7c shows the discharge and charge polarization curves with N-GPC-900 and commercial Pt/C as air cathodes. Compared to a Pt/C-driven Zn–air battery, a higher charge–discharge voltage gap was observed for a N-GPC-900-driven one. Remarkably, the N-GPC-900-driven Zn–air battery exhibited cycling stability confirmed by 200 charge/discharge cycles. The battery made with the obtained carbon catalyst showed a higher potential range when compared to the reference material (from 0.5 to 3 V). The N functional groups, embedded in the carbon structure under the influence of a high carbonization temperature, could be responsible for this. Due to similar electrochemical properties of both the obtained N-rich carbon materials and the commercial Pt/C catalyst, these new materials have considerable potential as metal-free catalysts.

## Materials and methods

### Preparation of N-doped carbon from gelatine and green algae

In the proposed method, nitrogen-rich porous carbon materials were prepared using a solution of (40 wt.%) colloidal silica (SiO_2_) as well as green algae (*Chlorella vulgaris*; nitrogen-rich porous carbon from green algae, N-APC) or gelatine (nitrogen-rich porous carbon from gelatine, N-GPC), at a weight ratio of 2.5:1. In the first step, green algae (5 g) were sonicated in distilled water in an ultrasonic bath for 1.5 h. Gelatine (1.5 g) was dissolved in distilled water at 80 °C. In the second step, the colloidal silica solution was added to the samples. The resulting mass was stirred continuously until the deionized water evaporated, then was carbonized at 800 °C or 900 °C under N_2_ flow for 1 h, at a ramp up of 3 °C min^−1^. After carbonization, N-rich carbon was retrieved via removal of the silicate template using a 15% hydrofluoric acid (HF) solution and repeated washing with deionized water. The synthesized materials were then dried at 120 °C for 24 h.

Samples were labelled N-APC-T and N-GPC-T, where: N-APC-N-rich denotes porous carbon obtained from green algae, N-GPC-N-rich denotes porous carbon obtained from gelatine, T marks the carbonization temperature of 800 °C or 900 °C, as 800 or 900, respectively.

### Preparation of N-doped hybrid carbon materials

In the proposed method, hybrid carbon materials were produced using a colloidal silica (SiO_2_) solution (40 wt.%), green algae (*Chlorella vulgaris*) or gelatine, and graphene, in a weight ratio of 2.5:1:1. In the first step, the samples were prepared using graphene nanoplatelets (Sigma Aldrich, 1.2 g), green algae (1.2 g), and 10 mL of ethyl alcohol (99%). They were afterwards sonicated in an ultrasonic bath for 1.5 h. In the case of samples using gelatine, graphene nanoplatelets (Sigma Aldrich, 1.5 g) were added to a gelatine solution (0.5% w/v) and stirred at 80 °C for 24 h. In the second step, a colloidal silica solution was added and the resulting mass was stirred using a magnetic stirrer until all deionized water evaporated from the sample. Then, it was carbonized under the flow of N_2_ at 800 °C or 900 °C for 1 h, at a heating rate of 3 °C min^−1^. Finally, the N-rich carbon was retrieved by removing the silicate template with a 15% HF solution, then washing with deionized water and filtering. The obtained materials were dried for 24 h at 120 °C.

Samples were labelled N-APC-GR-T and N-GPC-GR-T, where: N-APC-GR—N-rich porous carbon obtained from green algae and graphene, N-GPC-GR—N-rich porous carbon obtained from gelatine and graphene, T—carbonization temperature of 800 °C or 900 °C, marked as 800 or 900, respectively.

### Chemical characterization

Raman spectrum tests were carried out using a Renishaw InVia Raman analyser with a 532 nm wavelength laser (Renishaw Company, Gloucestershire, the United Kingdom) powered at 2 mW. The microscopic features of the as-prepared N-rich carbons were analysed using a HRTEM FEI on a Tecnai F20 X-Twin apparatus (Brno, Czech Republic) with an acceleration voltage of 200 kV. X-ray photoelectron spectra were measured with a PHI5000 Versa Probe II Scanning XPS Microprobe (Chigasaki, Japan) using monochromatic AlKα X-ray radiation (1486.6 eV) at 25 W. Survey scans were taken at 117.4 eV analyser pass energy and multiple high-resolution scans were taken at 46.95 eV. The C1s peak position was set to 284.6 eV as an internal standard. Specific surface areas were measured with an automated volumetric analyser (ASAP 2020 Plus, Micromeritics, Norcross, the United States of America) using N_2_ adsorption and desorption isotherms at 77 K. The samples were outgassed in a vacuum at 200 °C for 24 h prior to the gas sorption measurements. The BET surface area was calculated using experimental points at a relative pressure of p/p_0_ = 0.05–0.25. Pore size distribution (PSD) was calculated using the nonlocalized density functional theory (NLDFT) method.

### Electrochemical measurements

All electrochemical measurements were performed using a computer-controlled potentiostat (Autolab, PGSTAT128N, Netherland) with a typical three-electrode cell. A platinum wire was used as the counter electrode; a silver-silver chloride electrode (Ag/AgCl) saturated in a 3 M KOH solution was the reference; the obtained catalyst, deposited on a pre-polished glassy carbon electrode (GC) of a 5 mm diameter (disc geometric area 0.196 cm^2^) served as the working electrode. Preparing ink with the tested material was an uncomplicated process; it consisted of combining 2.5 mg of the produced carbon with deionized water, ethanol, and a Nafion solution (0.5 wt.% water solution) in an appropriate volume ratio of 8:2:1. ORR activity of a Pt/C catalyst (20 wt.% of Pt, Sigma Aldrich) was measured for comparison. An amount of the prepared ink was applied to the GC, such that the amount of catalyst equalled 0.4 mg cm^−1^, and was set aside for a few minutes to allow the volatile solvents to evaporate.

A flow of O_2_ and N_2_ was maintained over the electrolyte (0.1 M KOH) in order to ensure continued saturation during the recording of electrochemical measurements. The activity of the electrocatalysts was evaluated through CV and LSV techniques on RDE, using a glassy carbon electrode. The scan rate of CV measurements was kept at 10 mV s^−1^ in a potential range from 0 to 0.8 V, while that for LSV was 5 mV s^−1^ with a disc electrode rotating at 800 to 2800 rpm. The number of electrons (n) transferred in ORR was calculated based on LSV results, using Koutecky–Levich (K–L) equations^39–41^:

1 $$ {\text{J}}^{ - 1} = {\text{ J}}_{{\text{L}}}^{ - 1} + {\text{ J}}_{{\text{K}}}^{ - 1} = \, ({\text{B}}\omega^{1/2} )^{ - 1} + {\text{ J}}_{{\text{K}}}^{ - 1} $$

2 $$ {\text{B}} = 0.62{\text{nFC}}_{0} ({\text{D}}_{0} )^{2/3} \nu^{ - 1/6 } $$

Therein, J stands for measured current density, J_L_ for limiting current density, and J_K_ for kinetic current density; ω is defined as the angular velocity of the electrode; n is the number of electrons involved in a charge transfer in ORR; F is the Faraday constant (96,485 C mol^−1^); C_0_ is the bulk concentration of O_2_ (1.2 × 10^–6^ mol L^−1^ in 0.1 M KOH); D_0_ is the diffusion coefficient for oxygen (1.9 × 10^–5^ cm^2^ s^−1^ in 0.1 M KOH); ν defines the kinetic viscosity of 0.1 M KOH (0.01 cm^2^ s^−1^)^39–41^. Using both equations, it is possible to determine the slope of the K–L plot and consequently estimate the number of electron transfers in the oxygen reduction reaction.

### Zn–air battery measurements

Zn–air battery tests were performed on home-built electrochemical cells, where the cathode consisted of a gas diffusion layer loaded with catalysts (GDL) and the anode of a zinc plate. The cathode was prepared by pipetting catalyst ink onto carbon paper until loading of 0.71 and 2.13 mg cm^−2^ was achieved. To ensure reversible zinc electrochemical reactions at the anode, the electrolyte used in the battery was 6 M KOH containing 0.2 M ZnCl_2_. Nickel mesh was used as the current collector. All data were collected from the as-prepared cell with an electrochemical workstation (Autolab, PGSTAT128N, Netherland), at room temperature. A commercial Pt/C catalyst was used as the reference material.

## Conclusions

In summary, we have developed effective N-GPC and N-APC electrocatalysts by using cheap, natural materials like green algae and gelatine as precursors. These catalysts were produced by carbonizing nonporous precursors with a pore-forming agent in order to generate a hierarchical porous structure. The as-prepared, metal-free mesoporous carbons possessed a high density of N-containing active sites (N content 7.7 at.%) and a high specific surface area (880 m^2^ g^−1^). The main electrochemical techniques considered were CV and RDE. The N-rich porous carbons are effective electrocatalysts, as shown by both half-cell and full-cell measurements. They are promising replacements of the Pt-based ORR catalyst in alkaline Zn–air batteries. Importantly, the 3D mesoporous network structure was highly favourable for rapid ORR species transport. The ORR performance that N-GPC-900 exhibited was high compared to the state-of-the-art. This efficiency is promoted by the simultaneous presence of pyrrolic-N and graphitic-N sites, the amount of the latter being especially important in determining ORR activity. It further depends on carbonization temperature and greatly benefits from exposed graphitic-N along the outer and inner carbon sheets with a high surface area. This is the synergetic effect in our developed metal-free catalyst. The synthesis method presented here provides N-rich porous carbons that will be useful in applications such as catalysis, supercapacitors, fuel cells, and Zn–air batteries.
